# Reduction of Cardiovascular Risk Using Proprotein Convertase Subtilisin/Kexin Type 9 Inhibitors in Patients With Acute Coronary Syndrome: A Systematic Review

**DOI:** 10.7759/cureus.34648

**Published:** 2023-02-05

**Authors:** Ahmad M Rabih, Ahmad Niaj, Aishwarya Raman, Manish Uprety, Maria Jose Calero, Maria Resah B Villanueva, Narges Joshaghani, Nicole Villa, Omar Badla, Raman Goit, Samia E Saddik, Sarah N Dawood, Lubna Mohammed

**Affiliations:** 1 Internal Medicine, California Institute of Behavioral Neurosciences & Psychology, Fairfield, USA; 2 Obstetrics and Gynecology, California Institute of Behavioral Neurosciences & Psychology, Fairfield, USA; 3 Research, California Institute of Behavioral Neurosciences & Psychology, Fairfield, USA; 4 Psychiatry and Behavioral Sciences, California Institute of Behavioral Neurosciences & Psychology, Fairfield, USA; 5 General Surgery, California Institute of Behavioral Neurosciences & Psychology, Fairfield, USA; 6 Pediatrics, California Institute of Behavioral Neurosciences & Psychology, Fairfield, USA

**Keywords:** acute coronary syndrome, alirocumab, atherosclerotic cardiovascular disease, cardiovascular risk reduction, efficacy of pcsk9 inhibitors, evolocumab, major adverse cardiovascular events, pcsk9, pcsk-9 inhibitor, pcsk9 inhibitors safety

## Abstract

Proprotein convertase subtilisin/kexin type 9 (PCSK9) is a hepatic enzyme that regulates blood cholesterol levels by degrading low-density lipoprotein (LDL) receptors from the surface of hepatocytes. Studies have shown that inhibiting this molecule decreases the cardiovascular risk in individuals with atherosclerotic cardiovascular disease (ASCVD) by lowering low-density lipoprotein cholesterol (LDL-C). Two major cardiovascular outcome trials showed that the use of the PCSK9 inhibitors (alirocumab and evolocumab) in patients with recent acute coronary syndrome (ACS) is associated with a lower risk of further cardiovascular (CV) events. Information regarding the use of these monoclonal antibodies for primary prevention has also been reported by these trials. The goal of this systematic review is to describe the mechanism of PCSK9 inhibitors and further discuss their ability to reduce CV risk in high-risk populations. The search strategy was used in a systematic way using PubMed Central, Google Scholar, and ScienceDirect. We included randomized control trials (RCTs), systematic reviews, and narrative reviews in English published in the last five years. Observational studies, case reports, and case studies were excluded. The quality of the studies was evaluated using the Cochrane Collaboration Risk of Bias Tool, Assessment of Multiple Systematic Reviews 2, and Scale for the Assessment of Narrative Review Articles. A total of 10 articles were included in this systematic review. These included an RCT, a systematic review, and eight narrative reviews. Our study suggested that adding PCSK9 inhibitors to background statin therapy for selected patients with high-risk factors demonstrated substantial benefits in reducing overall CV morbidity and mortality after ACS. Multiple studies have demonstrated the short-term safety of low LDL-C levels caused by these drugs. However, long-term safety must be assessed with further studies.

## Introduction and background

According to the data collected by the Global Burden of Disease (GBD), acute coronary syndrome (ACS) remains a crucial cause of morbidity and mortality worldwide. In 2019, the number of cardiovascular (CV) cases grew to 523 million and fatalities to 18.6 million [1]. ACS refers to a variety of acute cardiological conditions, including ST-elevated myocardial infarction (STEMI), non-ST-elevated myocardial infarction (NSTEMI), and unstable angina (UA). Following ACS, the possibility of further major adverse cardiovascular events (MACE) still persists [2]. To tackle this issue, the American College of Cardiology/American Heart Association (ACC/AHA) guidelines now suggest using high-intensity statins or maximum-tolerated statin therapy to lower low-density lipoprotein cholesterol (LDL-C). Furthermore, in very high-risk individuals, it is considered to add ezetimibe to maximally tolerated statin therapy when the LDL-C level remains ≥70 mg/dL (≥1.8 mmol/L). If the LDL-C level remains ≥70 mg/dL (≥1.8 mmol/L), adding proprotein convertase subtilisin/kexin type 9 (PCSK9) inhibitors is reasonable to reduce the risk of further MACE [1,3]. Despite the use of statins for this purpose, there remains a substantial risk of developing MACE in high-risk groups. Additionally, the side effects of statins such as myalgia have limited their use in these patients [4].

In 2006, it was discovered that mutations in the PCSK9 gene led to higher LDL-C and cardiovascular disease (CVD) risk. This prompted further research in the field and ultimately, the discovery of novel PCSK9 inhibitors [5]. The use of these monoclonal antibodies (alirocumab and evolocumab) has changed the landscape for dyslipidemia treatment and lowered the cardiovascular risk after ACS. Additionally, over the last two years, major cardiovascular outcome trials (ODYSSEY OUTCOMES study and FOURIER study) have made it possible to reduce residual atherosclerotic cardiovascular disease (ASCVD) risk and reaffirmed the benefits of maintaining low LDL-C levels in high-risk subgroups [6].

Under physiological conditions, PCSK9 works by promoting lysosome-mediated low-density lipoprotein receptor (LDL-R) degradation. This results in decreased expression and recycling of the receptor causing a buildup of LDL-C in the circulation. By inhibiting PCSK9, these effects are reversed, resulting in lower levels of LDL-C in the blood. Inhibitors of the PCSK9 enzyme lower LDL-C levels by 40-60% in both statin-intolerant and statin-tolerant patients; however, there are no data on cardiovascular outcomes in the former group. When added to background statin medications, PCSK9 inhibitors significantly reduce MACE in individuals with chronic ASCVD or those with ACS [7]. This is due to the synergistic effect created by combining these medications, as statins inhibit hepatic cholesterol production and further lower LDL-C levels [5].

The FOURIER trial evaluated evolocumab, while alirocumab was studied in the ODYSSEY OUTCOMES trial. Treatment with these medications has achieved much lower plasma concentrations of LDL-C compared to previous lipid-lowering therapy [8]. This systematic review aims to discuss the implications of both the above-mentioned trials with regard to PCSK9 inhibitors, review their clinical use, as well as what the future holds for these drugs.

## Review

Methods

The Preferred Reporting Items for Systematic Reviews and Meta-Analyses (PRISMA) 2020 guidelines were implemented to describe the findings of this systematic review [9].

Eligibility Criteria

Studies with the following criteria were selected as eligible for the review: studies written in the English language, human studies, and free full-text articles published in the last five years (2017-2022). Further inclusion criteria encompass criteria of the ODYSSEY OUTCOMES trial (alirocumab and CVD outcomes), which includes patients who had ACS in the past 12 months with LDL-C levels of at least 70 mg/dL, a non-high-density lipoprotein (non-HDL) cholesterol level of at least 100 mg/dL, or an apolipoprotein B level of at least 80 mg/dL, and were receiving statin therapy at a high-intensity dose or at the maximum tolerated dose. The inclusion criteria followed by the FOURIER trial (evolocumab and CVD outcomes) were patients with one major or two minor cardiovascular risk factors, fasting LDL-C ≥ 70 mg/dL or non-HDL cholesterol ≥ 100 mg/dL, triglycerides ≤ 400 mg/dL, and taking statins. We restricted our choice of studies to randomized control trials (RCTs), narrative reviews, and systematic reviews. Observational studies, case reports, and case studies were excluded. Further exclusion criteria included paid articles and animal studies.

Databases and Search Strategy

A search was conducted through PubMed Central (MEDLINE), Google Scholar, and ScienceDirect. Keywords and Medical Subject Heading (MeSH) terms were used to identify all potentially relevant articles discussing the efficacy of alirocumab and evolocumab in reducing cardiovascular outcomes in patients with recent ACS. The search was performed using keywords such as PCSK9 inhibitors, alirocumab, evolocumab, cardiovascular risk, acute coronary syndrome, and myocardial infarction. The Boolean method was used to combine the keywords and MeSH terms to search through the various databases. Table 1 below shows the details of the search strategy that was used in this systematic review. All references were grouped and alphabetized using the EndNote reference manager for duplicate removal. The records were first reviewed based on the titles and abstracts, excluding irrelevant studies. Following this, full-text papers were reviewed for further exclusion.

**Table 1:** Details of the search strategy used in this systematic review PCSK9: proprotein convertase subtilisin/kexin type 9; MI: myocardial infarction; ACS: acute coronary syndrome.

Database	Search strategy	Filters applied	Results
PubMed	Cardiovascular risk OR myocardial infarction OR acute coronary syndrome OR ("myocardial infarction/drug therapy"[Majr] OR "myocardial infarction/mortality"[Majr] OR "myocardial infarction/prevention and control"[Majr]) AND PCSK9 inhibitors OR evolocumab OR alirocumab OR ("PCSK9 inhibitors/pharmacology"[Majr] OR "PCSK9 inhibitors/therapeutic use"[Majr])	Free full-text, last 5 years, humans, English, middle-aged, and 45+	231
Google Scholar	Keywords: Alirocumab, evolocumab, PCSK9 inhibitors, cardiovascular events, ACS, MI	2017-2022	250
ScienceDirect	Keywords: PCSK9 inhibitors, cardiovascular risk, ACS	2017-2022, open access and open archive	299

Risk of Bias in the Studies

The remaining 22 papers were thoroughly assessed by two individual authors separately for quality using study-specific tools. Each assessment tool has a specific scoring system and studies with a score of >70% were accepted to be used in this review. Table 2 below summarizes the quality assessment of the studies as well as the respective tools used.

**Table 2:** Details of quality assessment tools used to assess the studies in this systematic review CCRBT: Cochrane Collaboration Risk of Bias Tool; RCT: randomized control trial; AMSTAR 2: Assessment of Multiple Systematic Reviews 2; PICO: population, intervention, comparison, and outcomes; RoB: risk of bias; SANRA 2: Scale for the Assessment of Narrative Review Articles.

Quality assessment tool	Type of study	Items & their characteristics	Total score	Accepted score (>70%)	Accepted studies
CCRBT	RCT	Seven items: random sequence generation and allocation concealment (selection bias), selective outcome reporting (reporting bias), other sources of bias, blinding of participants and personnel (performance bias), blinding of outcome assessment (detection bias), and incomplete outcome data (attrition bias). Bias assessed as LOW RISK, HIGH RISK, or UNCLEAR.	7	5	Schwartz et al. (2018) [8]
AMSTAR 2	Systematic review	Sixteen items: (1) Did the research questions and inclusion criteria for the review include the components of PICO? (2) Did the report of the review contain an explicit statement that the review methods were established prior to the conduct of the review and did the report justify any significant deviations from the protocol? (3) Did the review authors explain their selection of the study designs for inclusion in the review? (4) Did the review authors use a comprehensive literature search strategy? (5) Did the review authors perform study selection in duplicate? (6) Did the review authors perform data extraction in duplicate? (7) Did the review authors provide a list of excluded studies and justify the exclusions? (8) Did the review authors describe the included studies in adequate detail? (9) Did the review authors use a satisfactory technique for assessing the risk of bias (RoB) in individual studies that were included in the review? (10) Did the review authors report on the sources of funding for the studies included in the review? (11) If meta-analysis was justified, did the review authors use appropriate methods for the statistical combination of results? (12) If meta-analysis was performed, did the review authors assess the potential impact of RoB in individual studies on the results of the meta-analysis or other evidence synthesis? (13) Did the review authors account for RoB in individual studies when interpreting/discussing the results of the review? (14) Did the review authors provide a satisfactory explanation for, and discussion of, any heterogeneity observed in the results of the review? (15) If they performed quantitative synthesis, did the review authors carry out an adequate investigation of publication bias (small study bias) and discuss its likely impact on the results of the review? (16) Did the review authors report any potential sources of conflict of interest, including any funding they received for conducting the review? Scored as YES or NO. A partial Yes was considered as a point.	16	12	Schmidt et al. (2017) [10]
SANRA 2	Narrative review	Six items: justification of the article’s importance to the readership, statement of concrete aims or formulation of questions, description of the literature search, referencing, scientific reason, and appropriate presentation of data. Scored as 0, 1, or 2.	12	9	Iannuzzo et al. (2021) [1], Schwartz et al. (2019) [2], Ferri et al. (2020) [3], Cho et al. (2020) [4], Lee et al. (2018) [5], Wong et al. (2019) [6], Diaz et al. (2021) [7], Kim et al. (2020) [11]

Results

Study Selection and Quality Assessment

**Figure 1:**
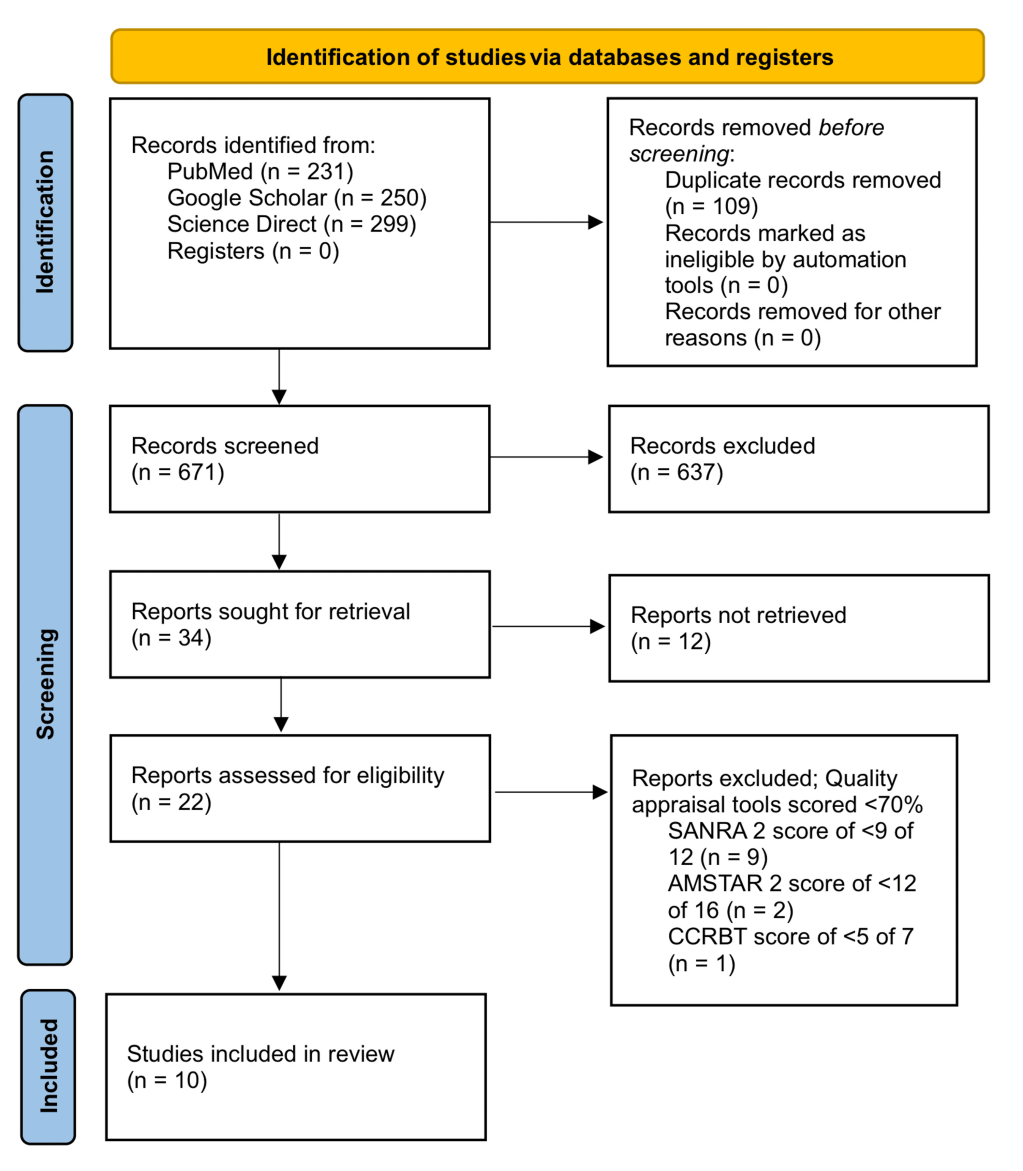
Flowchart demonstrating the selection process of the included articles SANRA 2: Scale for the Assessment of Narrative Review Articles 2; AMSTAR 2: Assessment of Multiple Systematic Reviews 2; CCRBT: Cochrane Collaboration Risk of Bias Tool.

Using three databases (PubMed, ScienceDirect, and Google Scholar), a total of 780 results were obtained. These results were grouped together to remove duplicates, which were 109 in number. Out of the 671 remaining papers, 637 were excluded on the basis of irrelevant titles and abstracts. There were 34 reports left, which were thoroughly screened as full-text papers. Out of those, 22 were excluded. Quality assessment was then performed on the remaining studies, using tools specific to each type of study. Ten final studies that scored >70% on the quality assessment tools were chosen to be included in this review. This consisted of one RCT, one systematic review, and eight narrative reviews. The last date of data collection was April 21, 2022. A flow diagram showing the details of the identification and screening process used to select the final articles for this review is presented in Figure 1.

Study Characteristics

The specific characteristics of the systematic and narrative reviews are summarized in Table 3 below.

**Table 3:** Main characteristics of the systematic review and the narrative reviews included in this review RCTs: randomized control trials; CVD: cardiovascular disease; PCSK9: proprotein convertase subtilisin/kexin type 9; MI: myocardial infarction; NR: not reported; ACS: acute coronary syndrome; MACE: major adverse cardiac events; ASCVD: atherosclerotic cardiovascular disease; LDL-C: low-density lipoprotein cholesterol.

First author, year	Study type	Total participants	Inclusion criteria	Outcomes & key points	Funding
Schmidt et al. (2017) [10]	Systematic review	60,997	RCTs were eligible if they included adults 18 years of age or older, with or without a history of CVD. Participants could have had normal lipid levels or hypercholesterolemia. No constrictions were applied to comorbidities	Compared to placebo, PCSK9 inhibitors (alirocumab and evolocumab) reduced the risk of CVD, MI, stroke (combination of ischemic and hemorrhagic events), and all-cause death (high-certainty evidence) (for alirocumab).	This study was funded by multiple institutions
Iannuzzo et al. (2021) [1]	Narrative Review	NR	NR	In post-ACS patients, PCSK9 inhibitor therapy reduces MACE significantly. This appears to be accomplished in two ways: directly by altering plaque composition and then stabilizing it, and indirectly by interfering with lipid metabolism and platelet aggregation. PCSK9 inhibitors improve endothelial function in patients with familial hypercholesterolemia, a well-known risk factor for cardiovascular disease.	No external funding
Schwartz et al. (2019) [2]		NR	NR	PCSK9 inhibition has been shown to lower MACE in patients with recent ACS.	NR
Ferri et al. (2020) [3]		NR	NR	PCSK9 inhibitors reduced MACE but did not clearly reduce mortality.	NR
Cho et al. (2020) [4]		NR	NR	In individuals with ASCVD, the PCSK9 monoclonal antibodies evolocumab and alirocumab provide further LDL-C reduction and reduce cardiovascular events in addition to background statin therapy.	Funded by multiple institutions
Lee et al. (2018) [5]		NR	Inclusion criteria of FOURIER, ODYSSEY, and SPIRE 1, 2	In individuals with ASCVD, PCSK9 inhibitors reduced LDL-C by 50% or more and reduced MACEs.	NR
Wong et al. (2019) [6]		NR	NR	PCSK9 inhibitors led to a significant reduction in LDL-C in patients with recent ACS according to many trials.	Received external funding
Diaz et al. (2021) [7]		18,924	Inclusion criteria of the ODYSSEY trial	According to the findings, statin intolerance is linked to a significantly increased cardiovascular risk in patients with recent ACS. Regardless of statin intolerance or statin intensity, MACE was lowered. For this group of patients, the availability of lipid-lowering medication with the PCSK9 inhibitor alirocumab provides an effective therapeutic alternative for reducing MACE.	Received external funding
Kim et al. (2020) [11]		NR	NR	PCSK9 inhibitors are a significant advancement in cholesterol treatment, lowering CVD events and LDL-C by 50% while maintaining tolerable discontinuation rates.	This article did not receive any funding

The characteristics of the RCT used in this review are summarized in Table 4 below.

**Table 4:** Main characteristics of the randomized control trial accepted in our systematic review RCT: randomized controlled trial; LDL-C: low-density lipoprotein cholesterol; HDL: high-density lipoprotein; apo-B: apolipoprotein-B; mg/dL: milligram per deciliter.

First author, year	Study type	Number of patients	Inclusion criteria	Age	Median duration of follow up	Outcomes & key points	Funding
Schwartz et al. (2018) [8]	RCT	18,924	Acute coronary syndrome in the previous 12 months, had an LDL-C level of at least 70 mg/dL, a non-HDL cholesterol level of at least 100 mg/dL, or an apo-B level of at least 80 mg/dL, and were receiving statin therapy at a high-intensity dose or at the maximum tolerated dose.	40 years or older	2.8 years	A total of 903 patients (9.5%) in the alirocumab group and 1052 patients (11.1%) in the placebo group experienced a composite primary end-point event (hazard ratio, 0.85; 95% CI, 0.78 to 0.93; P < 0.001). In the alirocumab group, 334 patients (3.5%) died, while 392 patients (4.1%) died in the placebo group (hazard ratio, 0.85; 95% CI, 0.73 to 0.98). Patients having a baseline LDL-C level of ≥100 mg/dL had a larger absolute benefit from alirocumab in terms of the composite primary end-point than those with a lower baseline level. Except for local injection-site reactions, the incidence of adverse events was identical in both groups (3.8% in the alirocumab group vs. 2.1% in the placebo group).	Received external funding

Discussion

This section will focus on the real-life efficacy and safety of PCSK9 inhibitors. It will also touch on PCSK9-related cardiovascular outcomes trials and their implications on patient care, health economics, and future perspectives of PCSK9 inhibitors.

Discovery of the PCSK9 Inhibitors

PCSK9 is a protease enzyme from the proteinase K family that is encoded by the PCSK9 gene found on chromosome one. By acting as a ligand, this enzyme attaches to low-density lipoprotein (LDL) receptors located on the cell membrane of liver cells and stimulates the lysosomal degradation of these receptors. Consequently, LDL-C uptake from the bloodstream is reduced [4]. This physiologic function was first understood when activating mutations in the protein resulted in hypercholesterolemia in a group of patients. In patients with familial hypercholesterolemia (FH) as well as the general population, these mutations limit the elimination of LDL-C in the plasma. As confirmed by various studies, high blood levels of LDL-C continue to be a significant stepping stone for the development of CVD [10]. On the other hand, the Dallas Heart Study found that 2.6% of 3,363 black individuals with inactivating mutations of PCSK9 had more desirable CVD outcomes due to lowered LDL-C levels. These results lay the theoretical groundwork for exploring a PCSK9-targeted intervention to decrease LDL-C [11].

Various pharmacological methods of inhibiting PCSK9 have been identified for cardiovascular risk reduction. In particular, two PCSK9 monoclonal antibodies (evolocumab and alirocumab) have shown promising results when given alongside statin therapy in patients with recent ACS [4]. Statins work by augmenting the production of both LDL receptors and PCSK9. Therefore, PCSK9 inhibition appears to be an appealing technique for enhancing statin therapy efficacy as well as reducing LDL-C.

Mechanism of PCSK9 Inhibitors

**Figure 2:**
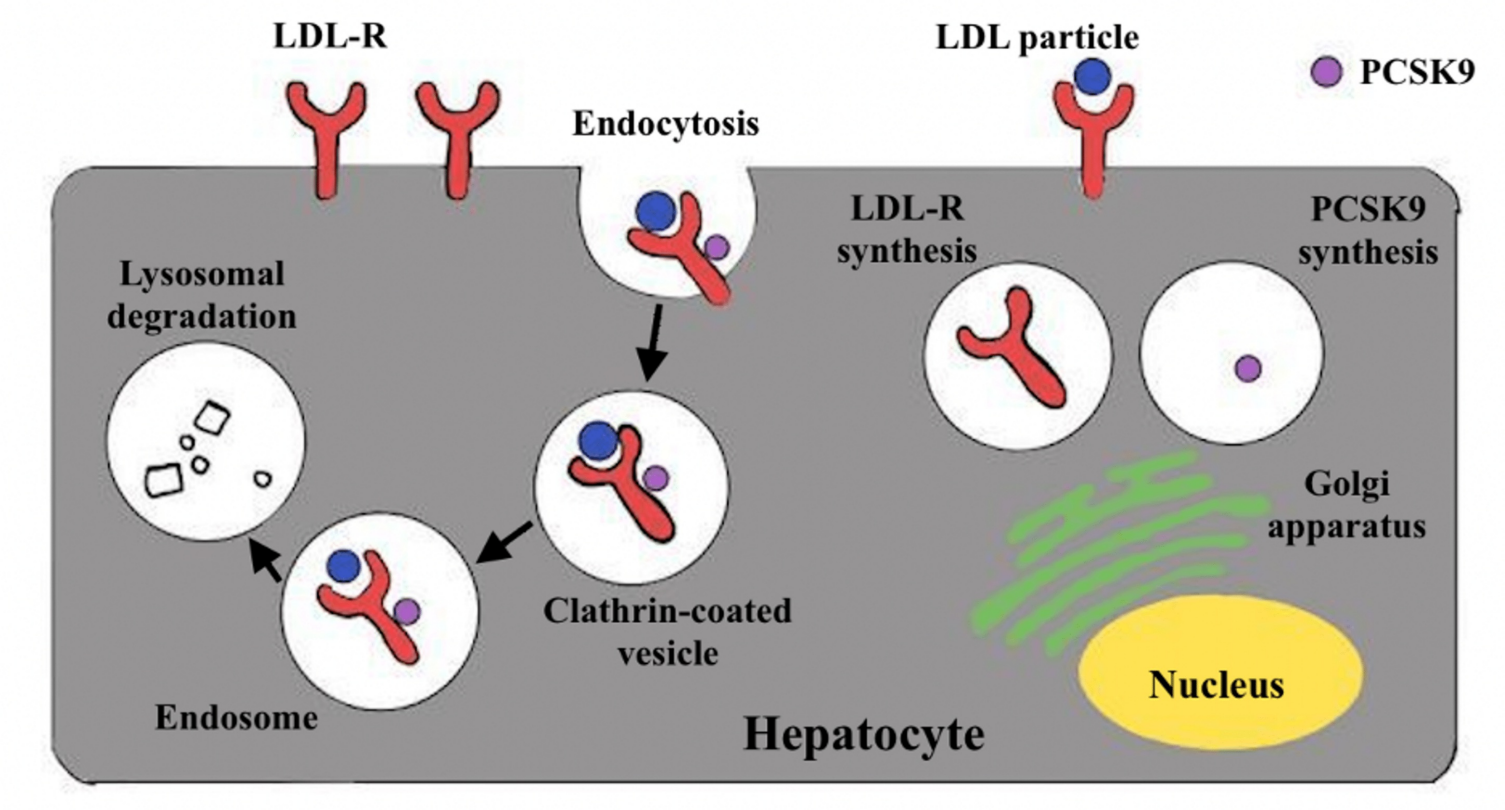
Diagram demonstrating the mechanism of how PCSK9 binds to the LDL-R and eventually leads to the lysosomal degradation of it PCSK9: proprotein convertase subtilisin/kexin type 9; LDL: low-density lipoprotein; LDL-R: low-density lipoprotein receptor. The figure is created by the first author Ahmad M. Rabih.

In the human body, LDL-C levels are regulated primarily through LDL receptors found on hepatocytes. As mentioned above, PCSK9 is a proteolytic enzyme that destroys LDL receptors and hence indirectly modulates serum LDL-C. Blocking or binding circulating PCSK9 using alirocumab or evolocumab leads to more LDL receptors and a decrease in serum LDL-C. Extrahepatic organs such as the kidney, gut, and central nervous system contribute to PCSK9 production and, presumably, local modulation of LDL-R expression, despite its hepatic origin. If PCSK9 attaches to the LDL-R before the LDL particle, the entire complex enters the hepatocyte and gets destroyed by the lysosome. This process is demonstrated in Figure 2 below. This implies that lowering the amount of free PCSK9 available to attach to the LDL-R will reduce the destruction of the receptor, therefore, resulting in less LDL-C in the plasma [12].

Efficacy and Safety of PCSK9 Inhibitors

Multiple phase III clinical trials have shown the effectiveness of alirocumab and evolocumab in reducing LDL-C levels, particularly in patients with familial hypercholesterolemia or with a history of ASCVD. Recently, there have been two large cardiovascular outcome trials that explained the effectiveness of PCSK9 inhibitors in reducing LDL-C levels [1]. These trials discovered that over the period of 12-52 weeks, evolocumab proved to lower LDL-C by 18.3% to 66%, whereas alirocumab lowered LDL-C by 36.3% to 61.0% over a period of 24-78 weeks. The FOURIER trial showed that evolocumab successfully reduced LDL-C by almost 60% over a period of 48 weeks in patients with recent ACS versus the placebo group. Whereas the ODYSSEY OUTCOMES trial concluded that alirocumab reduced LDL-C by almost 61% in patients who suffered from ACS in the last 12 months [8,13].

Multiple pooled studies and meta-analyses have been conducted to investigate whether alirocumab and evolocumab are safe to use clinically or not. One specific meta-analysis included results from almost 30 RCTs with around 45,500 patients. This study described no particular association between using PCSK9 inhibitors as a therapy and the development of neurocognitive adverse events, diabetes mellitus, or myalgia. Furthermore, there was no association with increased creatine kinase, aspartate aminotransferase, or alanine levels [11,13].

The FOURIER trial was one of the first studies to provide data about the long-term safety of evolocumab. Almost 27,500 patients with a history of ASCVD were studied for an average of 2.2 years. The only reported adverse event in the study group was injection site reactions. No other adverse events, such as neurocognitive reactions or development of diabetes mellitus, were reported [14]. The ODYSSEY OUTCOMES trial conducted a study on 18,900 patients, all of whom had ACS in the last 12 months, to evaluate the safety of alirocumab. Over a period of 2.8 years, the trial reported similar results to the FOURIER trial in regard to the injection site and neurocognitive adverse events. Furthermore, results from a big meta-analysis of 14 RCTs demonstrated that patients who used alirocumab had very low rates of neurocognitive side effects with an overall rate as low as <1% [5,13].

A humanized PCSK9 monoclonal antibody (bococizumab) was discovered recently to lower LDL-C. In six trials including approximately 4,000 subjects, a significant number of patients developed high titer anti-drug antibodies, which led to a significant decline in its cholesterol-lowering effect. This issue resulted in the removal of bococizumab from the market. However, compared to bococizumab, the other fully humanized monoclonal antibodies alirocumab and evolocumab were associated with a lower percentage of immunogenicity [8,15].

The Effects of PCSK9 Inhibitors on Cardiovascular Function

There are many reports on the effect of PCSK9 inhibitors on cholesterol regulation; however, only a few studies focused on their effect on cardiovascular function. It is a well-known fact that high levels of circulating LDL-C impose a major risk on the cardiovascular health of individuals. This new, alternative technique for reducing this risk in dyslipidemia patients, via PCSK9 inhibition, has proven to be more efficacious than regular statin therapy. LDL-C, inflammation, smooth muscle cell death, and aortic cholesteryl ester deposition were all reduced when PCSK9 was inhibited [4].

**Figure 3:**
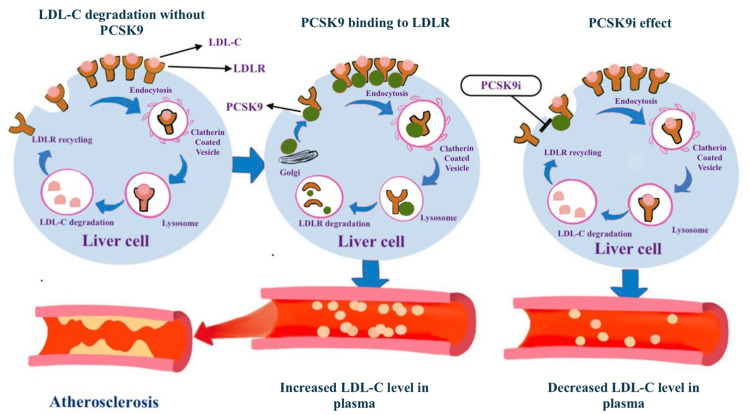
Diagram showing a summary of how PCSK9 inhibitors work. These drugs act by decreasing LDL-R degradation leading to better lipid metabolism and decreased plasma LDL-C, ultimately leading to a decrease in the risk of atherosclerosis PCSK9: proprotein convertase subtilisin/kexin type 9; PCSK9i: proprotein convertase subtilisin/kexin type 9 inhibitors; LDL: low-density lipoprotein; LDL-C: low-density lipoprotein cholesterol; LDL-R: low-density lipoprotein receptor. The figure is created by the first author Ahmad M. Rabih.

Furthermore, PCSK9 inhibitors enhance LDL-R levels, resulting in improved cholesterol metabolism as well as a reduction in inflammation and atherosclerotic lesion size. Figure 3 below shows a summary diagram highlighting the potential cardiovascular benefits of PCSK9 inhibitors. Despite mounting evidence that PCSK9 inhibitors can lower cholesterol levels and reduce the risk of cardiovascular disease, the basic processes underpinning PCSK9 inhibitors' effects on cardiac function have not been studied enough and it requires further research [16,17].

PCSK9 Cardiovascular Outcome Trials

Both evolocumab and alirocumab had their own individual cardiovascular outcomes trials, which will be discussed in this section. The first large RCT to discuss the use of these medications in high-risk patients was the FOURIER trial, which was published in March 2017. It is essential to understand the details of these studies to use these medications more extensively in the medical field. Table 5 below summarizes the two randomized controlled clinical trials studying the cardiovascular outcomes of PCSK9 inhibitors.

**Table 5:** Summary of the two randomized controlled clinical trials studying the cardiovascular outcomes of PCSK9 inhibitors * Death resulting from an acute MI, heart failure, stroke, CV procedures, CV hemorrhage, other CV causes, and sudden cardiac death. **Any death with a clear relationship to underlying CHD (death secondary to acute MI, sudden death, and heart failure). CV: cardiovascular; MI: myocardial infarction; PAD: peripheral artery disease; LDL-C: low-density lipoprotein cholesterol; mg/dL: milligram per deciliter; non-HDL-C: non-high-density lipoprotein cholesterol; UA: unstable angina; mg: milligram; SC: subcutaneously; CHD: coronary heart disease; ASCVD: atherosclerotic cardiovascular disease.

Variables	FOURIER	ODYSSEY OUTCOMES
Design	Randomized, double-blinded, placebo-controlled	
Median follow-up	2.2 years	2.8 years
Inclusion criteria	ASCVD (MI, non-hemorrhagic stroke, or symptomatic PAD), LDL-C level ≥70 mg/dL or non-LDL-C ≥100 mg/dL, while taking a maximum tolerated dose of statin therapy	Recent acute coronary syndrome (acute MI or UA) event within the last 12 months, LDL-C level ≥70 mg/dL, non-HDL-C ≥100 mg/dL, or apolipoprotein B ≥80 mg/dL, while taking a maximum tolerated dose of statin therapy
Intervention	Evolocumab 140 mg every two weeks or 420 mg every month	Alirocumab 75 or 150 mg SC every two weeks (adjusted in a blinded fashion to achieve LDL-C level of 25-50 mg/dL)
Primary endpoint	Major CV events (CV death, MI, stroke, hospitalization for UA, or coronary revascularization)	CHD death, non-fatal MI, ischemic stroke, or UA requiring hospitalization
Secondary endpoint	CV death, MI, or stroke	CHD event: CHD death, non-fatal MI, UA requiring hospitalization, or ischemia-driven coronary revascularization, non-fatal ischemic stroke, or CV death
Outcomes	CV death*, MI stroke (ischemic and hemorrhagic), UA requiring hospitalization, coronary revascularization	CHD death**, MI (non-fatal), stroke (fatal and non-fatal), UA requiring hospitalization
Conclusions	Evolocumab effectively reduced LDL-C levels by more than 50% when added to statin medication at 48 months. And it reduced the risk of primary efficacy endpoint by 15% as well as the composite of CV mortality, MI, and stroke by 20% in patients with stable ASCVD	Alirocumab showed a significant reduction of LDL-C levels by 55% when added to a statin medication. It reduced the risk of primary efficacy endpoint by 15% as well as all-cause death in patients with acute coronary syndrome within a year

FOURIER trial (evolocumab): At the start of the trial, around 70% of patients were taking high-dose statins, 80% had hypertension, and 36% were diabetics [18]. The participants' mean age was 62.5 years, with 75% of them being males. The baseline LDL-C level was 92 mg/dL in both the study and placebo groups [8,19]. After a 12-month follow-up period, the LDL-C level dropped to ≤70 mg/dL in around 87% of the patients, and to ≤25 mg/dL LDL-C in up to 42% of them [20]. In a prespecified secondary analysis of over 25,500 subjects with ASCVD, after four weeks of taking evolocumab, almost 90% of patients had low LDL-C levels. Among those, 10% reached LDL-C levels of <19 mg/dL and levels <10 mg/dL were achieved by 2% of the patients. Overall, the risk reduction of MACE in the patients taking evolocumab was 15%, especially in individuals who reached LDL-C levels as low as 10 mg/dL [21,22].

ODYSSEY trial (alirocumab): In this trial, 18,924 individuals who had ACS in the previous 12 months were randomly assigned to receive a single 75 mg dose of alirocumab or a placebo weekly. Over 90% of the enrolled patients were chosen because of their high (>70 mg/dL) LDL-C levels. Overall, this trial has shown the great potential of having alirocumab in the market. It demonstrated a massive 55% fall in LDL-C levels when given to patients on background statins [7,23,24]. Patients with higher (>100 mg/dL) baseline levels of LDL-C experienced an even greater fall in absolute risk reduction (ARR) of 3.4% for the primary endpoint, compared to those with lower baseline levels of LDL-C. Throughout the trial, therapy with alirocumab was also concluded to be well-tolerated and safe [25].

Upon analysis of the results of both trials, it has been concluded that the use of these drugs in addition to statin therapy led to significant reductions in LDL-C levels and a reduction in the risk of MACE. The FOURIER trial demonstrated that evolocumab reduced the risk of the primary endpoint by 15% and the secondary endpoint by 20%. The ODYSSEY trial showed that alirocumab significantly reduced the risk of the primary endpoint by 15% and reduced the risk of the key secondary endpoint by 16%, compared with the placebo. These results further establish the positive correlation between low LDL-C levels and reduced cardiovascular risk [25,26].

In clinical practice, these trials suggest that PCSK9 inhibitors can be used as an additional therapy in patients with ASCVD or ACS who are unable to achieve target LDL-C levels with statin therapy alone. These drugs are indicated for individuals with CVD, to lower their chances of developing further cardiovascular events, including myocardial infarction (MI) and stroke. Additionally, they are also given to reduce LDL-C levels of adult patients diagnosed with either a homozygous or a heterozygous FH. The drugs can be taken alone, with food, or with other lipid-lowering agents [5]. It is worth noting that these drugs are not without risk and cost; hence, the decision to prescribe them should be made on an individual basis, taking into account the patient's cardiovascular risk, other comorbidities, and the cost-benefit ratio.

PCSK9 Inhibitors’ Economic Value

Since their approval in 2015, evolocumab and alirocumab's poor cost-effectiveness has been a stumbling block to their broad adoption. In 2017, researchers used data from the FOURIER trial to construct a cost-effectiveness analysis model to analyze whether it would be wiser to combine these medications with statins, rather than using statins alone. With a PCSK9 inhibitor costing $14,300 per year, the additional cost of using one of these drugs plus a statin was $337,729 yearly. This amount is three times higher than the generally accepted cost of $100,000. This implies that the cost of these monoclonal antibodies would have to decrease by at least $5,459 yearly to meet the norm of societal acceptance. Similarly, the ODYSSEY OUTCOMES trial's cost-effectiveness analysis model concluded that the price of alirocumab would have to be lowered from $14,560 to $1,974 for the drug to be considered cost-effective. Practice models with the integration of specialty pharmacies have been proposed to optimize patient access to PCSK9 inhibitors [4,6].

Limitations

We faced a couple of limitations during our study that are worth mentioning. This study was limited to free full-text articles published in the last five years in the English language. Also, given that these medications are relatively new to the market, there were only two main clinical trials to draw information from. Nonetheless, due to the large sample sizes in both the FOURIER and ODYSSEY trials, we were able to draw fair conclusions regarding the efficacy and safety of PCSK9 inhibitors.

## Conclusions

The FOURIER and ODYSSEY OUTCOMES trials have shown that the usage of PCSK9 inhibitors in patients with ASCVD results in the reduction of LDL-C and MACE when given to patients taking statin therapy. PCSK9 inhibitors are indicated for use in high-risk patients who are unable to meet target LDL-C levels with high-dose statins and those who are intolerant to statins. The safety of these drugs (evolocumab and alirocumab) has also been assessed by these studies and is confirmed to be highly positive. Nevertheless, further research on the long-term safety of these drugs needs to be undertaken. Also, the safety and efficacy of these medications in different ethnic groups need to be evaluated. Further cardiovascular outcome trials regarding the use of PCSK9 inhibitors in the early stages of cardiovascular events are also essential. Lastly, the role of PSCK9 inhibitors in lowering LDL-C levels in high-risk groups has been established; however, further research is required in lower-risk populations.
