# Correction: Reduction of Cardiovascular Risk Using Proprotein Convertase Subtilisin/Kexin Type 9 Inhibitors in Patients With Acute Coronary Syndrome: A Systematic Review

**DOI:** 10.7759/cureus.c270

**Published:** 2025-08-26

**Authors:** Ahmad M Rabih, Ahmad Niaj, Aishwarya Raman, Manish Uprety, Maria Jose Calero, Maria Resah B Villanueva, Narges Joshaghani, Nicole Villa, Omar Badla, Raman Goit, Samia E Saddik, Sarah N Dawood, Lubna Mohammed

**Affiliations:** 1 Internal Medicine, California Institute of Behavioral Neurosciences & Psychology, Fairfield, USA; 2 Obstetrics and Gynecology, California Institute of Behavioral Neurosciences & Psychology, Fairfield, USA; 3 Research, California Institute of Behavioral Neurosciences & Psychology, Fairfield, USA; 4 Psychiatry and Behavioral Sciences, California Institute of Behavioral Neurosciences & Psychology, Fairfield, USA; 5 General Surgery, California Institute of Behavioral Neurosciences & Psychology, Fairfield, USA; 6 Pediatrics, California Institute of Behavioral Neurosciences & Psychology, Fairfield, USA

This article has been corrected to fix the following typo present in the PRISMA diagram:

"Duplicate records removed (n=**108**)" has been corrected to "Duplicate records removed (n=**109**)"

